# Draft Genome Sequence of Clinical Isolate USM026 of the Pathogenic Yeast Candida parapsilosis

**DOI:** 10.1128/mra.00839-22

**Published:** 2022-10-31

**Authors:** Dina Yamin, Wan Khairunnisa Wan Juhari, Nur Waliyuddin Hanis Zainal Abidin, Shuhaila Mat-Sharani, Azian Harun

**Affiliations:** a Department of Medical Microbiology and Parasitology, School of Medical Sciences, Universiti Sains Malaysia, Kubang Kerian, Kelantan, Malaysia; b Malaysian Node of the Human Variome Project, School of Medical Sciences, Universiti Sains Malaysia, Kubang Kerian, Kelantan, Malaysia; c Human Identification/DNA Unit, School of Health Sciences, Universiti Sains Malaysia, Kubang Kerian, Kelantan, Malaysia; d Biomedicine Program, School of Health Sciences, Universiti Sains Malaysia, Kubang Kerian, Kelantan, Malaysia; e Hospital Universiti Sains Malaysia, Kota Bharu, Kelantan, Malaysia; University of California, Riverside

## Abstract

Here, we report the draft genome sequence of a Candida parapsilosis clinical isolate (USM026) that was recovered from a blood sample from a patient who was treated for a catheter-related bloodstream infection (CRBSI). The draft genome is 12,839,916 bp in length, with 22,076,712 reads, 249 scaffolds, and 5,537 genes.

## ANNOUNCEMENT

Candida parapsilosis, a yeast belonging to the cellular organisms (Eukaryota superkingdom, Fungi kingdom, Dikarya subkingdom, Ascomycota phylum, Saccharomycotina subfamily, Saccharomycetes class, Saccharomycetales order, Debaryomycetaceae family, and *Candida* genus), is a major cause of candidemia worldwide (1). In this announcement, we present the draft genome sequence of a clinical isolate of C. parapsilosis. This will contribute to our understanding of the genetic variability among C. parapsilosis isolates and provide clues to genomic evolution. This isolate was recovered from a patient who had been admitted to Hospital Universiti Sains Malaysia (USM) (Kota Bharu, Kelantan, Malaysia) and later developed a catheter-related bloodstream infection (CRBSI). This work was approved by the Human Research Ethics Committee of USM (approval number JEPeM-USM-16040162).

The isolate was subcultured on a Sabouraud dextrose agar (SDA) plate and incubated at 37°C overnight. Pure colonies were harvested in the stationary phase. Genomic DNA (gDNA) was extracted using a conventional DNA extraction method, namely, the phenol-chloroform-isoamyl alcohol DNA extraction protocol (2). Molecular identification by sequencing of the internal transcribed spacer (ITS) region of ribosomal DNA was performed for species confirmation (3).

The DNA sample was fragmented by sonication to a size of 350 bp. DNA fragments were end polished, A-tailed, and ligated with full-length adaptors for Illumina sequencing. Library preparation with further PCR amplification was performed using the NEBNext Ultra DNA library preparation kit for Illumina (New England Biolabs, USA) according to the manufacturer’s protocol. PCR products were purified (AMPure XP system), and libraries were analyzed for size distribution with an Agilent 2100 Bioanalyzer and quantified using real-time PCR. The qualified libraries were used to carry out sequencing. The sequencing was performed using an Illumina NovaSeq 150-bp paired-end protocol. The original optic data obtained by high-throughput sequencing (Illumina platform) were transformed into raw sequence reads by CASAVA base calling and stored in FASTQ (fq) format. In total, the sequencing produced 22,076,712 raw paired-end reads. Quality control was performed using FastQC software, and the adapter and low-quality sequences were removed with Trimmomatic (v0.36) (4) by performing sliding window trimming with a minimum average quality score of 20 and a minimum sequence read length of 20 bases.

Genome assembly and annotation were performed in the Galaxy platform using default parameters unless stated otherwise (5). The gDNA was sequenced to an average sequencing depth of 254× using the Illumina NovaSeq 6000 platform, producing 2 × 150-bp paired-end reads. The genome sequences were assembled into contigs and scaffolds using SPAdes (v3.12.0) (6). The quality of the assembly was evaluated using QUAST (v5.0.2) (7). The final assembly for the C. parapsilosis USM026 strain consists of 249 scaffolds (≥1,000 bp), with a total length of 12,839,916 bp, a G+C content of 38.62%, an *N*_50_ value of 116,314 bp, and a longest scaffold of 343,659 bp. The assemblies align 98% against the reference genome, C. parapsilosis CDC317 (GenBank assembly accession number GCA_000182765.2), obtained from the NCBI GenBank database. Repetitive sequences of interspersed and low-complexity elements were masked using RepeatMasker (v4.0.9) (http://repeatmasker.org) with combined Dfam (v3.0) and RepBase (release 20181026) databases, based on fungal species, which resulted 2.28% masked bases (8). The final scaffolds were annotated using MAKER (v2.31.10) (9) with *ab initio* gene prediction using AUGUSTUS (v3.3.3) (10) and SNAP (v2013-11-29) (11), with soft masking in the repeat masking step. The training species was Candida albicans. C. parapsilosis strain USM026 was predicted to contain a total of 5,537 genes.

### Data availability.

This whole-genome shotgun project has been deposited in DDBJ/ENA/GenBank under BioProject accession number PRJNA610714 with GenBank accession number JADCQS000000000. The version described in this paper is the first version, JADCQS010000000. Raw reads are available in the NCBI Sequence Read Archive (SRA) under accession number SRR11249110.
